# Dual Role of Interleukin-20 in Different Stages of Osteoclast Differentiation and Its Osteoimmune Regulation during Alveolar Bone Remodeling

**DOI:** 10.3390/ijms24043810

**Published:** 2023-02-14

**Authors:** Bowen Meng, Benyi Yang, Yan Qu, Yuanbo Liu, Dongle Wu, Chaoran Fu, Yifan He, Xi Chen, Chufeng Liu, Xiaoxing Kou, Yang Cao

**Affiliations:** 1 Hospital of Stomatology, Sun Yat-sen University, Guangzhou 510055, China; 2 Guangdong Provincial Key Laboratory of Stomatology, Guangzhou 510055, China; 3 South China Center of Craniofacial Stem Cell Research, Guanghua School of Stomatology, Sun Yat-sen University, Guangzhou 510055, China; 4 Department of Orthodontics, Stomatological Hospital, Southern Medical University, Guangzhou 510260, China

**Keywords:** interleukin-20, osteoimmunology, osteoclast differentiation, signaling pathways, orthodontic tooth movement

## Abstract

Osteoimmunology mediators are critical to balance osteoblastogenesis and osteoclastogenesis to maintain bone homeostasis. A lot of the osteoimmunology mediators are regulated by interleukin-20 (IL-20). However, little is known about the role of IL-20 in bone remodeling. Here, we showed that IL-20 expression was correlated with osteoclast (OC) activity in remodeled alveolar bone during orthodontic tooth movement (OTM). Ovariectomize (OVX) in rats promoted OC activity and enhanced IL-20 expression, while blocking OC inhibited IL-20 expression in osteoclasts. In vitro, IL-20 treatment promoted survival, inhibited apoptosis of the preosteoclast at the early stages of osteoclast differentiation, and boosted the formation of osteoclasts and their bone resorption function at the late stages. More importantly, anti-IL-20 antibody treatment blocked IL-20-induced osteoclastogenesis and the subsequent bone resorption function. Mechanistically, we showed that IL-20 synergistically acts with RANKL to activate the NF-κB signaling pathway to promote the expression of c-Fos and NFATc1 to promote osteoclastogenesis. Moreover, we found that local injection of IL-20 or anti-IL-20 antibody enhanced osteoclast activity and accelerated OTM in rats, while blocking IL-20 reversed this phenomenon. This study revealed a previously unknown role of IL-20 in regulating alveolar bone remodeling and implies the application of IL-20 to accelerated OTM.

## 1. Introduction

Osteoimmunology is an emerging and interdisciplinary concept encompassing the interplay between the skeletal and immune systems in the bone turnover mechanism under physiology and pathology conditions [1]. The balance of bone remodeling is maintained with various cells, including osteocytes, osteoblasts, osteoclasts macrophages, T cells, and B cells [2,3,4], which derive from the same bone marrow micromilieu and share plenty of cytokines, receptors, transcription factors, and signaling pathways. An imbalance of bone and the immune system contributes to plenty of bone loss diseases, such as osteoporosis, periodontitis, and bone cracking in orthodontic tooth movement (OTM) [5,6,7]. Therefore, there is a need to find new alternative osteoclast-targeting agents for the treatment of bone loss diseases.

Several bone-affecting inflammatory cytokines such as TNF, IL-1, IL-6, IL-17, IL-22, IL-23 and IL-33, secreted by immune cells, synergistically act with RANKL and disrupt the balance of bone resorption and bone formation, leading to bone homeostasis disorders in bone loss diseases [7,8,9,10]. Interestingly, most of the above-mentioned cytokines are regulated by IL-20, one of the IL-20 subfamily cytokines that belongs to the IL-10 large family [11,12,13,14,15,16,17,18,19,20,21]. IL-20 is mainly produced by activated macrophages and skin cells [22,23]. Previous researchers have focused on the ability of IL-20 to act as a proinflammatory, chemotactic, and angiogenic cytokine in skin inflammation diseases [24], RA [16], liver fibrosis [14] and ischemic diseases [25] by activating its heterodimeric receptors (either IL-20RA/IL-20RB or IL-22RA1/IL-20RB). Recently, clinical trials and animal experiments have found elevated serum IL-20 levels in rheumatoid arthritis [16,26,27,28], osteoporosis [29], and cancer-related osteolytic diseases [30]. However, whether IL-20 contributes to bone loss diseases and whether IL-20 can serve as a potential target for bone remodeling is unclear.

OTM is involved in the complex biomechanical responses of periodontal tissue [31]. Mechanical force acts on the periodontal cells to produce a series of cytokines, including prostaglandins, IL-1, IL-6, and IL-17 [32,33], which initiate osteoclast procedures. Meanwhile, the receptor activator of nuclear factor (RANK)/RANK ligand (RANKL)/osteoprotegerin (OPG) and tumor necrosis factor (TNF)-α superfamily are increased in the periodontium. Thus, there is predominant clinical value and paramount scientific importance in identifying alternative osteoimmunology mediators targeting osteoclasts and treatments for bone-loss diseases. Our previous studies found that IL-20 was involved in osteoclastogenesis through the OPG/RANKL/RANK axis and the Notch pathway in vitro [34,35]. However, little is known about the role of IL-20 in orthodontic tooth movement and the specific downstream molecular mechanism of IL-20 in RANKL-induced osteoclastogenesis.

In this study, we explored the roles of IL-20 in regulating osteoclast fate in vitro and in vivo. We found that IL-20 promoted osteoclastogenesis through NF-κB-mediated signaling pathways. Moreover, blocking IL-20 decreased alveolar bone remodeling and orthodontic tooth movement, which indicated that IL-20 is a promising direction for the targeted regulation of osteoclastogenesis.

## 2. Results

### 2.1. Osteoclasts and IL-20 Were Synchronously Activated in OTM

**Figure 1:**
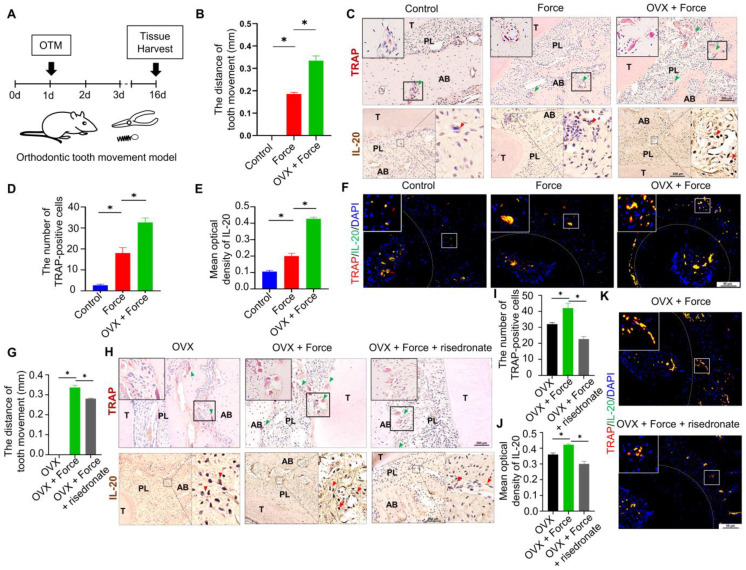
IL-20 accelerated OVX-induced bone loss in orthodontic tooth movement by promoting osteoclastogenesis. (**A**) Scheme illustrating the establishment of orthodontic tooth movement. (**B**) The distance of orthodontic tooth movement in the Control group, Force group, and OVX + Force group on day 16. (**C**) TRAP and Immunohistochemical staining showed that TRAP and IL-20 -positive cells and in the Control group, Force group, and OVX + Force group. Green triangles showed TRAP-positive osteoclasts. Red triangles showed IL-20-positive cells. T: tooth, PL: periodontal ligament, AB: alveolar bone. (**D**) The quantification of TRAP-positive osteoclasts in the Control group, Force group, and OVX + Force group. (**E**) Immunohistochemical staining and semiquantification of IL-20 in the Control group, Force group, and OVX + Force group. (**F**) Double-labelled immunofluorescence staining showed that, in the context of orthodontic force, the expression levels of IL-20 and osteoclast marker protein TRAP increased in the first molar periodontal ligament. (**G**) The distance of orthodontic tooth movement in the OVX group, OVX + Force group, and OVX + Force + risedronate group on day 16. (**H**) TRAP and immunohistochemical staining showed that TRAP and IL-20 -positive cells and in the OVX group, OVX + Force group, and OVX + Force + risedronate group. (**I**) The quantification of TRAP-positive osteoclasts in the OVX group, ovariectomy + Force group, and Ovariectomy + Force + risedronate group. (**J**) Immunohistochemical staining and semiquantification of IL-20 in the OVX group, OVX + Force group, and OVX + Force + risedronate group. (**K**) Immunofluorescence staining showed that the expression levels of IL-20 and osteoclast marker protein TRAP increased in the first molar periodontal ligament. * *p* < 0.05 vs. the control group. *n* = 6.

To explore the relationship between IL-20 and osteoclasts during alveolar bone remodeling, we constructed the rat OTM model (Figure 1A and Figure S1A). After mechanical force application for 16 days, the tooth movement distance increased to 0.18 mm. As shown by TRAP staining and immunohistology staining, the number of IL-20-positive cells and TRAP-positive osteoclasts simultaneously increased (Figure 1A–D and Figure S1D). To confirm whether IL-20 was expressed by the osteoclast, we used double immunofluorescent staining to show that IL-20 was colocalized with osteoclast markers RANK and TRAP (Figure 1C,E,F and Figure S1B,D,E). To further confirm whether IL-20 was synchronously activated with the osteoclast, we constructed the rat ovariectomize (OVX) model and applicated mechanical force. Compared to the Force group, the OVX + Force group increased tooth movement distance to 0.33 mm, accompanying an increased number of IL-20-positive cells colocalized with TRAP and RANK-positive osteoclasts (Figure 1A–F and Figure S1B,D,E). Additionally, immunofluorescence staining showed that IL-20^+^ and CD11b^+^ double-positive cells were observed in the alveolar bone of OTM mice, and OVX + Force treatment enhanced the numbers of IL-20 and CD11b-positive cells, suggesting that macrophages expressed IL-20 and participated in osteoclast activation (Figure S1C). In addition, compared to the OVX group, mechanical force enhanced the distance of tooth movement and simultaneously increased the number of IL-20-positive cells and TRAP-positive osteoclasts (Figure 1G–J). Moreover, risedronate, an anti-osteoporosis drug, decreased the distance of tooth movement in OVX rats and simultaneously decreased the number of IL-20-positive cells and TRAP-positive osteoclasts (Figure 1G–J). Synchronously, immunofluorescence showed that IL-20 colocalized with osteoclast markers TRAP and RANK was inhibited (Figure 1K and Figure S1B,F,G). Taken together, these results suggested that IL-20 was synchronously activated with osteoclasts in OTM, and may play a key role in osteoclastogenesis.

### 2.2. IL-20 Promoted Preosteoclast Proliferation by MAPK Pathways

**Figure 2:**
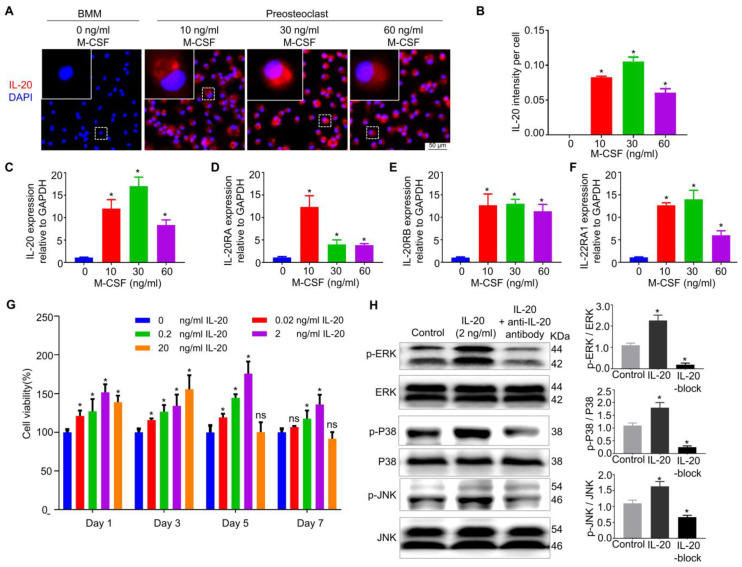
IL-20 promoted preosteoclast viability via MAPK pathway at the early stage of osteoclast differentiation. Cell viability was examined in M-CSF-induced preosteoclasts by a CCK8 assay. (**A**,**B**) The expression of IL-20 in M-CSF-induced preosteoclasts was determined by cellular immunofluorescence staining on day 2. (**C**–**F**) The mRNA expression levels of IL-20, IL-20RA, IL-20RB, and IL-22RA1 in preosteoclasts were evaluated by qRT-PCR after 2 days of M-CSF treatment. (**G**) Cell viability detection in preosteoclasts treated with a gradient of concentrations of IL-20 on days 1, 3, 5, and 7. (**H**) Preosteoclasts were treated with 2 ng/mL IL-20 or anti-IL-20 antibody. The levels of phosphorylation of proteins in the IL-20-mediated signaling pathway, including p38, phos-pho-p38, ERK, phospho-ERK, JNK, and phospho-JNK, were analyzed using Western blotting. IL-20-block group meant that cells were treated with IL-20 and anti-IL-20 antibody. * *p* < 0.05 vs. the 0 ng/mL IL-20 group. ns *p* > 0.05 vs. the 0 ng/mL IL-20 group. *n* = 6.

To identify whether IL-20 was expressed and to identify its function in osteoclast differentiation, the BMMs were induced into preosteoclasts and stained using cellular immunochemistry and immunofluorescence staining. Here, the immunofluorescence staining results revealed that different concentrations of M-CSF promoted IL-20 expression in preosteoclasts, and the cellular fluorescence intensity of IL-20 was highest after treatment with 30 ng/mL M-CSF (Figure 2A,B). Moreover, the qRT-PCR results showed that M-CSF differentially regulated the expression of IL-20 and its receptors IL-20RA, IL-20RB, and IL-22RA1 in preosteoclasts (Figure 2C–F). Additionally, we found that the expression of IL-20 in BMMs time-dependently increased with the treatment of 30 ng/mL M-CSF (Figure S2F). What is more, the cellular immunochemistry further confirmed that IL-20 and its receptors were expressed in preosteoclasts (Figure S2A). To explore the effect of IL-20 on preosteoclasts, we added different concentrations of IL-20 and detected preosteoclast function with CCK8 and flow cytometry. We found that IL-20 promoted the proliferation of preosteoclasts in a dose-dependent manner, and simultaneously suppressed preosteoclasts apoptosis (Figure 2G and Figure S2B–E). Western blotting suggested that IL-20 could perform a function by activating MAPK pathways, including ERK, p38, and JNK pathways. Moreover, blocking IL-20 with an anti-IL-20 antibody could partly inhibit the above-mentioned pathways’ activation (Figure 2H). In conclusion, IL-20 was expressed in MCF-induced preosteoclasts and promoted its function by the MAPK pathway.

### 2.3. IL-20 Had No Effect on Osteoclasts Differentiation and Functions at the Early Stage of Osteoclast Differentiation

**Figure 3:**
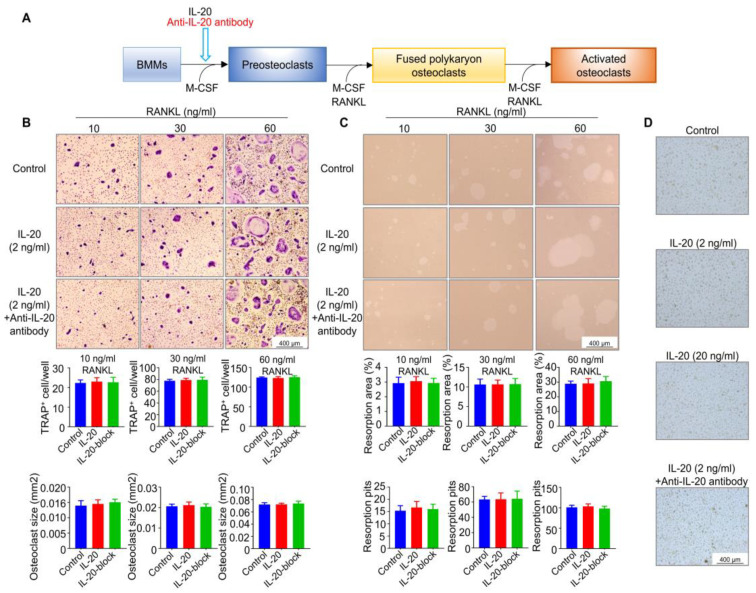
IL-20 had no effect on osteoclast formation at the early stage of osteoclast differentiation. (**A**) Scheme illustrating bone marrow-derived macrophages were cultured in osteoclast medium containing M-CSF and IL-20 or anti-IL-20 antibody at the early stage of osteoclast differentiation, and then induced with the presence of 10, 30, or 60 ng/mL RANKL. The control group included M-CSF-induced preosteoclasts induced with 10, 30, or 60 ng/mL RANKL. (**B**) TRAP staining was performed, and the number and size of TRAP-positive osteoclasts with more than three nuclei were quantified on day 6. IL-20-block group meant that cells were treated with IL-20 or anti-IL-20 antibody. (**C**) A bone resorption pit assay was performed to detect osteoclast function, and bone resorption pits were counted, and the area and number of bone resorption were quantified on day 6. IL-20-block group meant that cells were treated with IL-20 and anti-IL-20 antibody. (**D**) M-CSF-induced preosteoclasts were cultured in osteoclast medium containing different concentrations of IL-20 or anti-IL-20 antibody without RANKL. *n* = 6.

To prove the possibility of an interaction between IL-20 and osteoclastogenesis at the early stage of osteoclast differentiation from BMMs to preosteoclasts, we cultured BMMs with M-CSF (30 ng/mL) and IL-20 or anti-IL-20 antibody for 3 days and then changed to osteoclast medium without IL-20 for 6 days (Figure 3A). The TRAP staining and bone resorption experiments results revealed that there was no significant difference in TRAP-positive osteoclasts and bone resorption function compared to that of the respective control groups (Figure 3B,C). To further explore whether RANKL was essential for IL-20 regulation of osteoclastogenesis, we treated preosteoclasts with IL-20 without RANKL. The results of TRAP staining showed that IL-20 alone could not stimulate the formation of TRAP-positive cells (Figure 3D), which suggested that IL-20 was incapable of independently inducing osteoclastogenesis. Therefore, IL-20 had no effect on RANKL-induced osteoclast formation at the early stage of osteoclast differentiation.

### 2.4. IL-20 Promoted Osteoclasts Differentiation and Functions at the Late Stage of Osteoclast Differentiation

**Figure 4:**
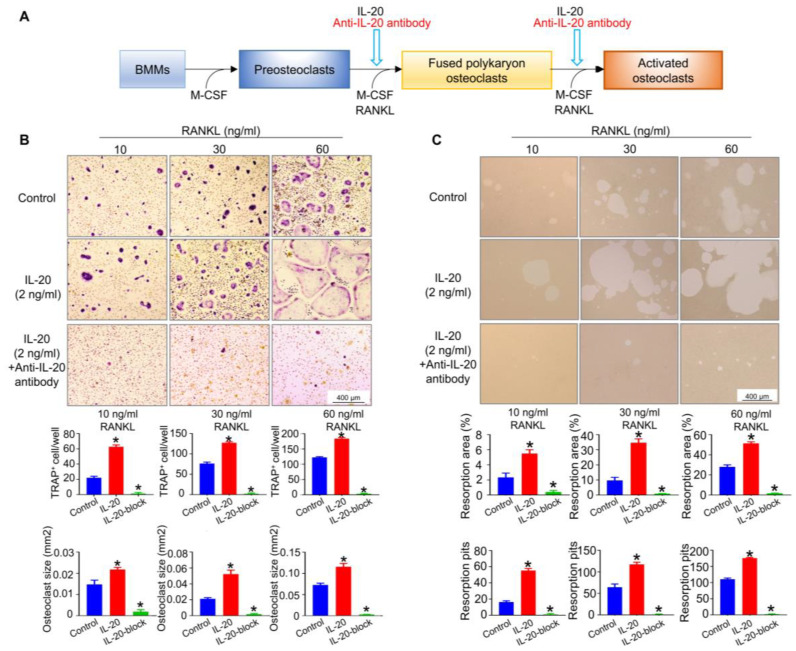
IL-20 promoted RANKL-induced osteoclast differentiation and bone resorption function. (**A**) Scheme illustrating M-CSF-induced preosteoclasts were cultured in osteoclast medium containing IL-20 or anti-IL-20 antibody at the late stage of osteoclast differentiation in the presence of 10, 30, or 60 ng/mL RANKL. (**B**) TRAP staining was performed, and the number and size of TRAP-positive osteoclasts with more than three nuclei were quantified at the late stage of differentiation on day 6. The control group included M-CSF-induced preosteoclasts induced with 10, 30, or 60 ng/mL RANKL. IL-20-block group meant that cells were treated with IL-20 and anti-IL-20 antibody. (**C**) A resorption pit assay was performed to detect osteoclast function, and resorption pits were counted at the late stage of differentiation on day 6, and the area and number of bone resorption were quantified at the stage of differentiation. IL-20-block group meant that cells were treated with IL-20 and anti-IL-20 antibody. * *p* < 0.05 vs. the control group. *n* = 6.

To identify the role of IL-20 in osteoclastogenesis at the late stage of differentiation, preosteoclasts were cultured in an osteoclast medium containing IL-20. After 6 days, the number and size of mature osteoclasts were determined by TRAP staining (Figure 4A). Compared with the control group, IL-20 promoted osteoclastogenesis in both numbers of TRAP+ cells and size with the increase in RANKL concentration, while IL-20-block using anti-IL-20 antibody could eliminate the promotion effects, even in a high concentration of RANKL (Figure 4B). Resorption assays showed the same pattern as TRAP staining and exhibited an increased bone resorption area and pits which were blocked with anti-IL-20 antibody (Figure 4C). Therefore, IL-20 promoted RANKL-induced osteoclast differentiation and bone resorption function.

### 2.5. IL-20 Promoted Osteoclast Differentiation through the NF-κB Pathway

**Figure 5:**
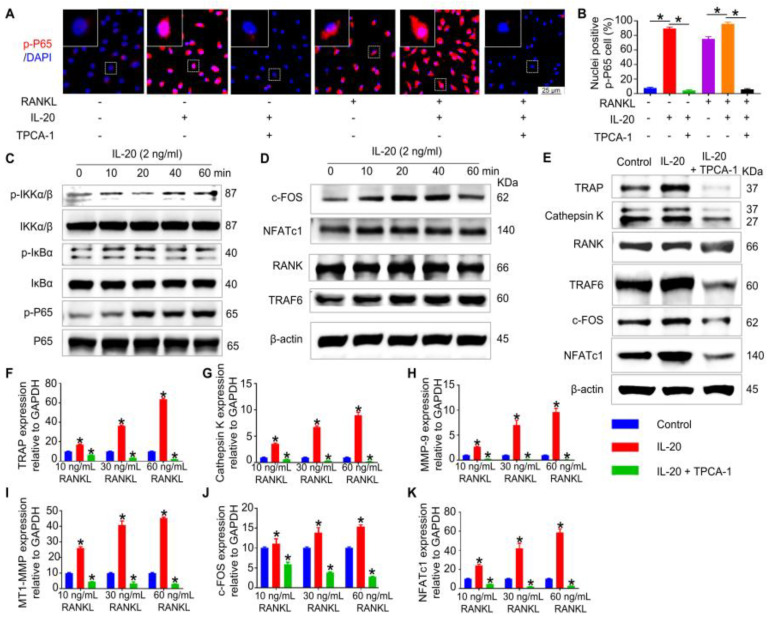
IL-20 promoted the expression of osteoclast-specific genes and proteins via NF-κB pathway during RANKL-induced osteoclast differentiation. Preosteoclasts were stimulated with IL-20 or NF-κB pathway inhibitor TPCA-1. (**A**,**B**) IL-20 promoted phospho-P65 nuclear translocation and was blocked by TPCA-1 in preosteoclasts after 1 h treatment, with or without RANKL. (**C**) The levels of phosphorylation for proteins in the NF-κB pathway, including the IKKα/β, IκB-α, and P65 proteins in preosteoclasts were detected using Western blotting. (**D**) The levels of activated proteins in preosteoclasts, including the RANK, TRAF6, c-Fos, and NFATc1 proteins without RANKL were detected using Western blotting. (**E**) The protein expression levels of TRAP, Cathepsin K, RANK, TRAF6, c-Fos and NFATc1 in osteoclasts were examined using Western blotting after 6 days of IL-20 treatment. (**F**–**K**) The mRNA expression levels of TRAP, Cathepsin K, MMP9, MT1-MMP, c-Fos, and NFATc1 in osteoclasts were detected by qRT-PCR after 6 days of IL-20 treatment. * *p* < 0.05 vs. the 0 ng/mL IL-20 group. *n* = 6.

To determine the molecular mechanism of IL-20 in osteoclastogenesis, immunofluorescence staining, and Western blotting, assays were performed. We found that IL-20 or RANKL alone promoted the expression of p-P65 and its translocation to the nucleus (Figure 5A,B). Furthermore, a combination treatment of IL-20 and RANKL could further enhance RANKL-induced activation of p-P65 (Figure 5A,B). Therefore, the results suggested that IL-20 was involved in osteoclast differentiation by a synergistic reaction with RANKL. In addition, TPCA-1, a NF-κB pathway inhibitor, notably suppressed the activation of p-NF-κB in preosteoclasts (Figure 5A,B). As the time of IL-20 treatment went by, Western blotting demonstrated that IL-20 promoted the phosphorylation of NF-κB cascade pathway, including IKKα/β, IκBα and NF-κB pathway (Figure 5C). To test the activation of the upstream and downstream pathways of the OPG/RANKL/RANK axis, Western blotting was performed and showed that downstream pathways TRAF6, c-FOS, and NFATc1 were activated at different times of IL-20 treatment. However, in upstream pathways, RANK showed no significant change (Figure 5D). However, TPCA-1 could partly block the activation of these pathways (Figure S3A,B). Similarly, after 6 days of IL-20 treatment, osteoclast-related proteins such as TRAP, Cathepsin K, TRAF6, c-FOS, and NFATc1 in RANKL-induced osteoclasts were verified and all upregulated by IL-20, and suppressed by TPCA-1 (Figure 5E and Figure S3C). To further confirm the results, qPCR has been used to examine the expression of osteoclastic genes with a gradient concentration of RANKL, and results indicated that IL-20 promoted osteoclastic gene expression via the NF-κB signaling pathway, including TRAP, Cathepsin K, MMP9, MT1-MMP, c-Fos, and NFATc1 (Figure 5F–K). Taken together, IL-20 may promote osteoclast differentiation and function by the NF-κB pathway.

### 2.6. Exogenous Injection of IL-20 Accelerated Tooth Movement in OTM

**Figure 6:**
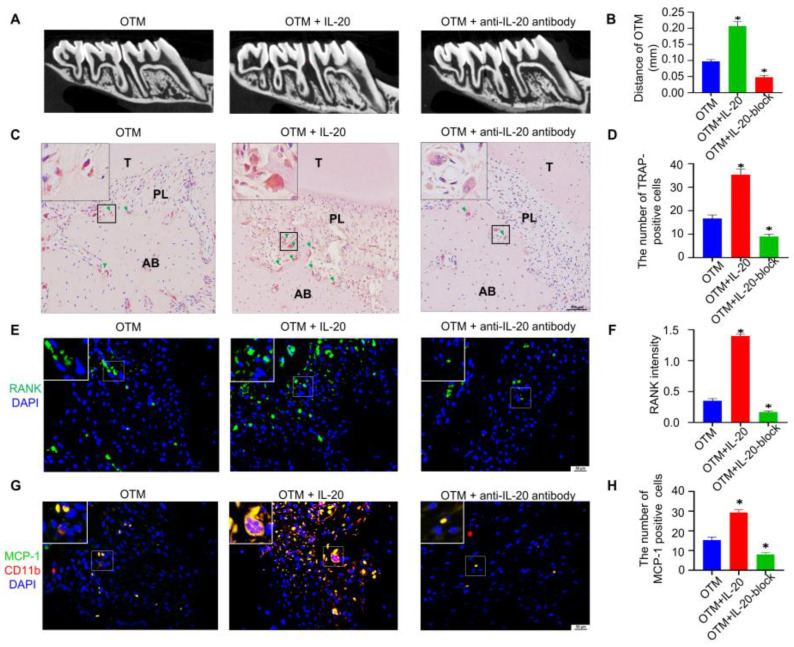
Therapeutic effect of IL-20 on orthodontic tooth movement. (**A**,**B**) Micro-CT showed the distance of orthodontic tooth movement between first and second molars treated with local infusion of IL-20 or an-ti-IL-20 antibody, seven days after the application of orthodontic force. OTM + IL-20-block group meant that rats were locally infused with anti-IL-20 antibody. (**C**,**D**) TRAP staining and the quantification of TRAP-positive osteoclasts in the OTM group, OTM + IL-20 group, and OTM + anti-IL-20 antibody group. Green triangles showed TRAP-positive osteoclasts. OTM + IL-20-block group meant that rats were locally infused with anti-IL-20 antibody. (**E**,**F**) Immunofluorescence staining showed that the expression levels of osteoclast marker protein RANK in the first molar periodontal ligament after the application of orthodontic force. OTM + IL-20-block group meant that rats were locally infused with anti-IL-20 antibody. (**G**,**H**) Immunofluorescence staining showed that the expression levels of MCP-1 and CD11b in the first molar periodontal ligament after the application of orthodontic force. * *p* < 0.05 vs. the control group. *n* = 6.

To demonstrate the potential clinical effects of IL-20, we locally infused IL-20 or anti-IL-20 antibody after the application of orthodontic force. HE staining verified the success of the rat model (Figure S4A). Micro CT manifested that local injection of IL-20 could increase the distance of OTM to 0.21 mm compared to the OTM group (Figure 6A,B). As shown by TRAP staining, local injection of IL-20 increased the number of TRAP-positive osteoclasts (Figure 6C,D). Additionally, immunofluorescent staining showed that IL-20 treatment increased the number of RANK-positive osteoclasts (Figure 6E,F). Moreover, local injection of anti-IL-20 antibody decreased the distance of OTM, TRAP-positive osteoclasts, and RANK-positive osteoclasts (Figure 6A–F). Monocyte chemoattractant protein-1 (MCP-1) is one of the key chemokines that regulates infiltration of monocytes/macrophages [36]. A previous study showed that IL-20 controlled T cell infiltration by regulating the expression of MCP-1 in mouse psoriasis models [19]. Thus, we investigated whether IL-20 affects MCP-1 expression and CD11b-positive macrophage infiltration in mice alveolar bone during OTM. Immunofluorescence staining showed that both MCP-1 staining, and CD11b-positive macrophages were observed in the alveolar bone of OTM mice, and IL-20 treatment enhanced the numbers of MCP-1 and CD11b-positive cells (Figure 6G,H). Moreover, local injection of anti-IL-20 antibody repressed IL-20 induced MCP-1 and CD11b expression in OTM mice (Figure 6G,H and Figure S4B). These results indicated that IL-20 may promote macrophage infiltration via upregulation of MCP-1. The results suggested that IL-20 could accelerate orthodontic tooth movement by activating osteoclasts (Figure S5).

## 3. Discussion

In this study, we discovered for the first time that IL-20 is involved in orthodontic tooth movement and may promote osteoclastogenesis, leading to accelerated orthodontic tooth movement in ovariectomy-induced rats. What is more, IL-20 and its receptors targeted M-CSF-induced preosteoclasts, and IL-20 promoted preosteoclast survival and inhibited apoptosis by MAPK pathways. Moreover, IL-20 promoted the osteoclast formation and bone resorption function induced by different concentrations of RANKL at the late stages of osteogenic differentiation, but had no effect at the early stages. Anti-IL-20 antibody notably blocked IL-20-induced osteoclastogenesis at the late stages. With respect to the molecular mechanisms, IL-20 activated the NF-κB signaling pathways and p-P65 translocation to the nucleus in the presence or absence of RANKL, and subsequently activated c-Fos and NFATc1. Finally, IL-20 promoted the expression of osteoclast-specific genes and proteins, including TRAP, CK, MMP9, MT1-MMP, c-Fos, NFATc1, and RANK.

It is well known that the survival factor M-CSF regulates the proliferation and apoptosis of BMMs and induces their differentiation into preosteoclasts through its receptor c-Fms [37,38]. In the bone marrow, osteoclasts are derived from granulocyte–macrophage progenitors (CFU-GM) of hematopoietic origin, and gene mutation of M-CSF completely inhibits both macrophage and osteoclast differentiation and formation [39,40]. However, Liu et al. reported that IL-20 specifically enhanced the proliferation, cell cycling, and colony formation of multipotential progenitors (CFU-GEMM) [41]. In addition, recent research has shown that IL-20, a potent angiogenic, chemotactic, and proinflammatory molecule, targets endothelial cells and epithelial cells and regulates their proliferation and apoptosis through related signaling pathways and genes, such as MAPK, JAK/STAT3, caspase 9 [42,43]. In our experiment, we found that IL-20 and its receptors were secreted by M-CSF-induced preosteoclasts in an autocrine manner, and that IL-20 promoted the proliferation and inhibited apoptosis of M-CSF-induced preosteoclasts in a dose-dependent manner. These findings suggested that IL-20 promoted the proliferation and inhibited apoptosis of M-CSF-induced preosteoclasts at the early stages of osteoclast differentiation.

In addition to M-CSF, RANKL has been shown to be at the forefront of osteoimmunology as another factor that is both necessary and sufficient for mature osteoclast formation, and it controls the level of bone resorption by binding to its specific receptor RANK [7,44,45,46,47]. RANKL is secreted not only by bone mesenchymal stem cells (BMSCs) or osteoblasts to sustain osteoclastogenesis by direct cell contact in vitro [48,49], but also by activated immune T cells that are involved in pathological bone loss [50,51]. RANKL–RANK binding subsequently activates the key transcription factors c-Fos and NFATc1, and finally leads to the expression of osteoclast marker molecules, such as TRAP, CK, MMP9, and MT1-MMP, that degrade the bone collagen and mineral matrix [52]. Previous studies showed that certain proinflammatory cytokines, such as TNF, IL-1, IL-6, and IL-22, were also involved in pathologic bone loss via synergistic action with RANKL [8,10,53]. In this study, we showed that IL-20 promoted the expression of RANK in response to RANKL and further contributed to the differential expression of c-Fos, NFATc1, TRAP, CK, MMP9, and MT1-MMP, which differentially regulated RANKL-induced osteoclast formation and bone resorption pits in vitro. Thus, we speculated that IL-20 was a downstream regulator of the OPG/RANKL/RANK axis, which mediated osteoclastogenesis by targeting preosteoclasts.

RANKL binding to its receptor RANK leads to the recruitment of the main adaptor molecule TRAF6, which activates downstream signaling pathways, including MAPK (p38, ERK, JNK), AKT and NF-κB. These pathways control osteoclast survival, apoptosis, and differentiation, and the functions of bone degradation and resorption [52]. Activation of p38 leads the transcriptional regulator mi/Mitf to enter the nucleus, where it regulates the gene expression of TRAP and CK [54]. The JNK pathway promotes formation of the activator protein-1 (AP1), which includes c-Fos and c-Jun, by activation of c-Jun phosphorylation [55]. In addition to M-CSF, RANKL can also activate the ERK and AKT pathways, which regulate the survival and proliferation of preosteoclasts and cytoskeletal rearrangement and motility in mature osteoclasts [52,56]. The NF-κB pathway is activated by the degradation of IκB proteins, which is induced by IκB kinase (IKK). Both the P65 and c-Fos transcription factors of the activator protein-1 (AP1) component are crucial for the activation of NFATc1, which controls the expression of osteoclast-specific genes, as mentioned above. In this study, we further found that IL-20 activated the phosphorylation of IKKα/β, IκB-α, P65, p38, ERK, and JNK by activating TRAF6 without RANKL, which ultimately resulted in the activation of c-Fos and NFATc1. Collectively, these results indicated that IL-20 activated TRAF6-mediated downstream NF-κB signaling pathways, and differentially induced preosteoclast differentiation into mature osteoclasts to perform bone resorption by synergistic action with RANKL, showing that NF-κB activation was not sufficient but indispensable for osteoclastogenesis.

Several proinflammatory cytokines are involved in osteoporosis. Neutralizing them with specific blocking molecules, including soluble receptors and antibodies, represents a significant therapeutic strategy for preventing bone loss in osteoporotic conditions [57,58,59,60]. Previous studies showed that the level of IL-20 was increased in osteoporotic patients and OVX-induced osteoporotic patients [29]. Moreover, soluble IL-20RA receptor blocked IL-20-induced osteoclast formation and inhibited the pathophysiology of bone loss in collagen-induced arthritis (CIA) [16]. In this study, we found that anti-IL-20 antibody inhibited IL-20-induced osteoclastogenesis at the late stages. Moreover, we found that IL-20 promoted osteoclastogenesis, leading to accelerated orthodontic tooth movement in OVX-induced rats. Risedronate, as a clinical drug to target osteoporosis, partly decreased the level of IL-20 and inhibited IL-20-induced osteoclast formation. Additionally, in our previous study, we found that risedronate inhibited orthodontic tooth movement by regulating the OPG/RANKL/RANK axis in ovariectomized rats [61]. Fortunately, specific IL-20 antibody can inhibit osteoclastogenesis and promote osteoblastogenesis. Moreover, IL-20 affected bone formation and downregulated osteoblastogenesis on osteoblasts; whereas, IL-20 antibody increased bone formation during fracture healing [62]. Our previous studies found that IL-20 differentially regulated the expression of OPG and RANKL in BMSCs and inhibited the osteogenic differentiation of MC3T3-E1 cells via the GSK3β/β-catenin signaling pathway [34,63]. It has the therapeutic potential to decrease osteoporotic bone loss and increase bone mineral density [29,62]. These findings suggested that blocking IL-20 might be a promising direction for targeted regulation in bone loss diseases.

Overall, this study elucidates the downstream mechanism of IL-20 in different stages of osteoclast differentiation and function in vitro, and the detailed function of how IL-20 participates in bone remodeling by regulating osteoclast differentiation and the osteoimmune microenvironment in vivo. Therefore, targeting IL-20 maybe a promising direction for the treatment of bone remolding-related diseases. Inhibition of IL-20 may repress osteoclast activity to ameliorate bone loss, such as in osteoporosis. On the other hand, IL-20 local injection may activate osteoclasts to accelerate the tooth movement process for orthodontic patients.

## 4. Materials and Methods

### 4.1. Animals

All Sprague-Dawley rats were purchased from the Animal Experimental Center of Guangzhou University of Chinese Medicine. The diet, housing conditions, and maintenance of rats were in accordance with the Institutional Animal Care and Use Committee (IACUC) of Sun Yat-sen University. All conducted experiments were approved by the Animal Ethical and Welfare Committee of Sun Yat-sen University (SYSU-IACUC-2018-000099, Guangzhou, China), which was approved on 10/25/2019. The rats were randomly divided into 7 groups (*n* = 6 in each group): group 1 (sham), group 2 (sham + Force), group 3 (ovariectomy), group 4 (ovariectomy + Force), group 5 (ovariectomy + risedronate + Force), group 6 (IL-20 + Force), and group 7 (anti-IL-20 + Force).

### 4.2. The Rat Model of Orthodontic Tooth Movement and Ovariectomy

The female rats were anesthetized for the bilateral ovariectomy operation. Briefly, we anesthetized the rats, removed the hair, disinfected the site, made 1 cm back incisions to remove the ovaries, and closed the incisions. After 1 week of postoperative recovery, 15 μg/kg risedronate was injected intraperitoneally in group 4 every 3 days for 3 weeks, while the other groups were given an equal volume of saline or drug.

Four weeks after ovariectomy, the rat models of orthodontic tooth movement were established as described in our published articles [61]. Under general anesthesia, we placed an orthodontic device consisting of nickel–titanium coiled springs and stainless steel (Xinya, Hang Zhou, China) between the incisors and the first molars on the maxilla, which caused the first molars to move medially. After self-etching using a dental etching agent (Heraeus Kulzer GmbH, Hanau, Germany), orthodontic devices were bonded with a chemically cured resin (3M Unitek, Sao Paulo, MN, USA) to provide 50 g orthodontic forces. On day 16, we measured the distance of orthodontic tooth movement and sacrificed the rats to collect the bilateral maxillary for the subsequent analysis.

### 4.3. Immunohistochemistry and TRAP Staining In Vivo

For immunohistochemistry, the maxillaries containing the molars were treated as described. Briefly, the decalcified samples were embedded for cutting, deparaffinized, and rehydrated, and endogenous peroxidase activity was blocked. Then, the slides were blocked, incubated with the primary and secondary antibodies, and stained with the chromogenic agent DAB and Meyer’s hematoxylin. For TRAP staining, we stained TRAP+ multinucleated osteoclasts with a TRAP staining kit (#G1050-50T, Servicebio, Wuhan, China), and counterstained the nucleus with hematoxylin.

### 4.4. Osteoclastogenesis Assays In Vitro

Osteoclasts were generated by the methods previously described [64,65]. Primary rat bone marrow macrophages (BMMs) from the whole bone marrow were isolated from the femur and tibia cavities of 4-week-old Sprague-Dawley rats. Rats were sacrificed after anesthesia; then, we flushed the bone marrow cavity with α-MEM, and purified it with RBC lysis buffer (CWBIO, Beijing, China) to collect the cell pellet. The cells were resuspended in 3 mL of α-MEM culture medium and incubated at 37 °C in a 5% CO_2_ incubator overnight. Then, we transferred the supernatant to a 15 mL centrifuge tube to collect the unattached cells (BMMs) the next morning. At the early stage of osteoclastogenesis, we treated BMMs with 30 ng/mL M-CSF (#400-28, Peprotech, Rocky Hill, NJ, USA) at 37 °C in a 5% CO_2_ incubator for 2–3 days to generate preosteoclasts, with or without IL-20 (#80422-RNAE, Sino Biological Inc., Beijing, China) or anti-IL-20 antibody (#80187/80453-R08H, Sino Biological Inc., Beijing, China). At the late stage of osteoclastogenesis, RANKL (#9366-TN-025, R&D Systems, Minneapolis, MN, USA) was added to the osteoclast medium containing IL-20 or anti-IL-20 antibody. The medium was replaced every 2 days until the formation of mature multinuclear osteoclasts was observed.

### 4.5. TRAP Staining In Vitro

According to the manufacturer’s protocol, TRAP staining was used to assess mature osteoclasts with an Acid Phosphatase Leukocyte (TRAP) Kit (#CS0740, Sigma, MO, USA) in vitro. After 6 days of osteoclastogenic differentiation, we counted TRAP-positive multinucleated cells with three or more nuclei as mature osteoclasts under an inverted fluorescence microscope (Zeiss, Jena, Germany). Six microscopic images were randomly taken at 50× magnification from three samples, and the average number was counted.

### 4.6. Resorption Pit Assay

A resorption pit assay was performed to detect the resorption function of osteoclasts. We cultured M-CSF-induced preosteoclasts in an Osteo Assay Surface multiple-well plate (Corning, New York, NY, USA), which was coated with an inorganic crystalline material on the surface. Briefly, mature osteoclasts were stripped with sodium hypochlorite, washed with distilled water and air-dried so that resorption pits could be easily observed with an inverted fluorescence microscope (Zeiss, Jena, Germany).

### 4.7. Cellular Immunochemistry and Immunofluorescence Staining

For cellular immunochemistry and immunofluorescence staining, preosteoclasts were cultured with 1 × 10^^6^ cells/well in 6-well plate containing glass slips and 5 × 10^^5^ cells/well in a laser confocal dish in an aseptic environment, respectively. When the cells covered 70–80% of the surface of the coverslips, we fixed the coverslips with 4% paraformaldehyde, permeabilized the cells with 0.2–0.25% Triton X-100, and blocked the cells with 3% BSA. Then, we incubated the cells with primary and secondary antibodies (anti-rabbit). Subsequently, we stained preosteoclasts with the chromogenic agent DAB and counterstained the cell nucleus with Meyer’s hematoxylin for immunocytochemistry or DAPI for immunocytofluorescence. Finally, glass coverslips were mounted with neutral balsam or antifluorescence quenching agent. An LSM780 confocal microscope (Zeiss, Germany) or an inverted fluorescence micro-scope (Zeiss, Germany) was used to capture cell images.

### 4.8. Cell Viability and Cytotoxicity Assay

Cell viability and proliferation were investigated using the Cell Counting Kit-8 (#CK04, CCK-8, Kumamoto, Dojindo, Japan). Briefly, we treated M-CSF-induced preosteoclasts with a gradient of concentrations of IL-20 (0–20 ng/mL) in 96-well plates (4000 cells/well). The medium was changed every 2 days. Then, we washed cells with PBS and added 100 µL of FBS-free DMEM containing 10 μL of WST-8 solution per well, and the cells were incubated for 1 h without light for the indicated times. Finally, we detected the absorbance at 450 nm using a microplate reader (Tecan SUNRISE microplate reader, Shanghai, Tecan, Männedorf, Switzerland).

### 4.9. Flow Cytometry Detection of Cell Apoptosis

Cell apoptosis was detected with an Annexin V-APC/7-AAD apoptosis kit (#AT105, MultiSciences Bio-tech, Hangzhou, China) by flow cytometry. Preosteoclasts were treated with various concentrations of IL-20 (0–20 ng/mL). According to the manufacturer’s protocol, we obtained the cells via trypsinization, washed the cells with PBS, and stained the cells with an apoptosis kit. After careful analysis with a Cytoflex flow cytometer (Beckman, Brea, CA, USA), four cell populations with apoptotic staining were distinguished as early apoptotic cells (APC+ 7-AAD-), late apoptotic cells (APC+ 7-AAD+), and viable cells (APC- 7-AAD-).

### 4.10. qRT-PCR Analysis

Briefly, we extracted total mRNA from 2 × 10^6^ cells/well in 6-well plate using an RNA-Quick Purification Kit (#ES-RN001, ES Science, Shanghai, China). PrimeScriptTM RT Master Mix (#RR047, Perfect Real Time) (Takara, Osaka, Japan) was used to obtain cDNA. The primers used for this study were commercially synthesized by Takara, and their sequences are listed in Table 1. Then, we quantified gene transcript levels with SYBR^®^ Premix Ex Taq™ (Tli RNaseH Plus) (Takara, Japan) in a QuantStudio 5 or 7 Flex Real-Time PCR System (Applied Biosystems™, Foster City, CA, USA) and analyzed the results using the 2^−△△CT^ method.

### 4.11. Western Blotting Analysis

After the treatment of IL-20, anti-IL-20 antibody, or TPCA-1 (MCE, Monmouth Junction, NJ, USA), we obtained protein from the samples with cold RIPA containing 1% protease inhibitor cocktail and 1% phosphatase inhibitors. Then, we detected the total concentration of sample proteins with a BCA Protein Assay Kit (CWBIO, Beijing, China). For Western blotting, we added 50 µg of sample protein per lane and then transferred the sample protein to a PVDF membrane. Subsequently, we blocked the membranes with TBST containing 5% BSA and incubated them with primary and secondary antibodies. Primary antibodies were specific for IKKα/β (#11930/8943, Cell Signaling Technology Inc., Beverly, MA, USA), phospho-IKKα/β (#2697, Cell Signaling Technology Inc., Beverly, MA, USA), IκBα (#4814, Cell Signaling Technology Inc., Beverly, MA, USA), phospho-IκBα (#2859, Cell Signaling Technology Inc., Beverly, MA, USA), P65 (#8242, Cell Signaling Technology Inc., Beverly, MA, USA), phospho-P65 (#3033, Cell Signaling Technology Inc., Beverly, MA, USA), p38 (#8690, Cell Signaling Technology Inc., Beverly, MA, USA), phos-pho-p38 (#4511, Cell Signaling Technology Inc., Beverly, MA, USA), JNK (#9252, Cell Signaling Technology Inc., Beverly, MA, USA), phospho-JNK, ERK (#4695, Cell Signaling Technology Inc., Beverly, MA, USA), phospho-ERK (#4370, Cell Signaling Technology Inc., Beverly, MA, USA), and c-Fos (#2250, Cell Signaling Technology Inc., Beverly, MA, USA); TRAP (#ab191406, Abcam, Cambridge, MA, USA), Cathepsin K (CK, #ab300569, Abcam, Cambridge, MA, USA), NFATc1 (#ab253477, Abcam, Cambridge, MA, USA), and TRAF6 (#ab33915, Abcam, Cambridge, MA, USA); and RANK (#sc-374360, Santa Cruz Biotechnology, Santa Cruz, CA, USA). The secondary antibodies were HRP Affinipure goat anti-rabbit IgG and HRP Affinipure goat anti-mouse IgG (#EM35111-01, EMAR, Beijing, China).

### 4.12. Statistical Analysis

All results in this study are presented as the mean ± standard deviation, with values from at least 3 or 6 independent experiments in vitro or in vivo, respectively. Using Student’s *t*-test or one-way ANOVA with Tukey’s post hoc analysis, statistically significant differences with values of *p* < 0.05 were determined by GraphPad Prism 7.04 software.

## Figures and Tables

**Table 1:** Primers sequences used for real-time PCR.

Gene	Forward Primer Sequence (5′-3′) (Tm) Reverse Primer Sequence (5′-3′) (Tm)	Product Size
IL-20	ACTGCAAACCTACAGGCGATACAA (64.1 °C) AGAACCTCACTAGATGGCGGAGA (63.7 °C)	163 bp
IL-20RA IL-20RB	GGGTCTACACGGAGTCGAAGTCA (64.9 °C) ACGCTCATAGTCCGAGGTCTCAA (64.5 °C) AGCACTTGATGGGTTAACAGCC (61.1 °C) AAAACAGAGACACAGCCCTCC (60.2 °C)	139 bp 72 bp
IL-22RA1	CCTACACGTGCCGAGTGAAGA (63.6 °C) AAAGCTCAGGACACGCTGGA (63.4 °C)	176 bp
Cathepsin K	CGGCTATATGACCACTGCCTTC (63.0 °C) TTTGCCGTGGCGTTATACATACA (64.3 °C)	114 bp
TRAP	TGGCAATGTCTCGGCACAA (64.9 °C) AGCATCACGGTGTCCAGCATAA (65.0 °C)	138 bp
MT1-MMP	GAGAACTTCGTGTTGCCTGATGAC (64.5 °C) TTTCTGGGCTTATCTGGGACAGAG (64.9 °C)	134 bp
MMP9	CATGCGCTGGGCTTAGATCA (64.6 °C) GAGGCCTTGGGTCAGGTTTAGAG (64.5 °C)	148 bp
NFATc1	CAAGTCTCACCACAGGGCTCACTA (64.0 °C) TCAGCCGTCCCAATGAACAG (62.2 °C)	144 bp
c-Fos	CGTCTTCCTTTGTCTTCACCTACC (64.8 °C) TTGCTGCTGCTGCCCTTT (63.7 °C)	81 bp
GAPDH	GGCACAGTCAAGGCTGAGAATG (64.4 °C) ATGGTGGTGAAGACGCCAGTA (62.8 °C)	143 bp

## Data Availability

Not applicable.

## Supplementary Materials

The following supporting information can be downloaded at https://www.mdpi.com/article/10.3390/ijms24043810/s1.

## References

[1] Okamoto K., Nakashima T., Shinohara M., Negishi-Koga T., Komatsu N., Terashima A., Sawa S., Nitta T., Takayanagi H. (2017). Osteoimmunology: The Conceptual Framework Unifying the Immune and Skeletal Systems. Physiol. Rev..

[2] Manolagas S.C., Jilka R.L. (1995). Bone marrow, cytokines, and bone remodeling. Emerging insights into the pathophysiology of osteoporosis. N. Engl. J. Med..

[3] Redlich K., Smolen J.S. (2012). Inflammatory bone loss: Pathogenesis and therapeutic intervention. Nat. Rev. Drug Discov..

[4] Weitzmann M.N., Ofotokun I. (2016). Physiological and pathophysiological bone turnover—Role of the immune system. Nat. Rev. Endocrinol..

[5] Arron J.R., Choi Y. (2000). Bone versus immune system. Nature.

[6] Takayanagi H., Ogasawara K., Hida S., Chiba T., Murata S., Sato K., Takaoka A., Yokochi T., Oda H., Tanaka K. (2000). T-cell-mediated regulation of osteoclastogenesis by signalling cross-talk between RANKL and IFN-gamma. Nature.

[7] Tsukasaki M., Takayanagi H. (2019). Osteoimmunology: Evolving concepts in bone-immune interactions in health and disease. Nat. Rev. Immunol..

[8] Kim K.-W., Kim H.-R., Park J.-Y., Oh H.-J., Woo Y.-J., Park M.-K., Cho M.-L., Lee S.-H. (2011). Interleukin-22 promotes osteoclastogenesis in rheumatoid arthritis through induction of RANKL in human synovial fibroblasts. Arthritis Rheum..

[9] Schulze J., Bickert T., Beil F.T., Zaiss M.M., Albers J., Wintges K., Streichert T., Klaetschke K., Keller J., Hissnauer T.-N. (2010). Interleukin-33 is expressed in differentiated osteoblasts and blocks osteoclast formation from bone marrow precursor cells. J. Bone Miner. Res..

[10] O’Gradaigh D., Ireland D., Bord S., Compston J.E. (2004). Joint erosion in rheumatoid arthritis: Interactions between tumour necrosis factor alpha, interleukin 1, and receptor activator of nuclear factor kappaB ligand (RANKL) regulate osteoclasts. Ann. Rheum. Dis..

[11] Rich B.E., Kupper T.S. (2001). Cytokines: IL-20—A new effector in skin inflammation. Curr. Biol..

[12] Li H.-H., Hsu Y.-H., Wei C.-C., Lee P.-T., Chen W.-C., Chang M.-S. (2008). Interleukin-20 induced cell death in renal epithelial cells and was associated with acute renal failure. Genes Immun..

[13] Hsu Y.-H., Wei C.-C., Shieh D.-B., Chan C.-H., Chang M.-S. (2012). Anti-IL-20 Monoclonal Antibody Alleviates Inflammation in Oral Cancer and Suppresses Tumor Growth. Mol. Cancer Res..

[14] Chiu Y.-S., Wei C.-C., Lin Y.-J., Hsu Y.-H., Chang M.-S. (2014). IL-20 and IL-20R1 antibodies protect against liver fibrosis. Hepatology.

[15] Huang K.-Y., Lin R.-M., Chen W.-Y., Lee C.-L., Yan J.-J., Chang M.-S. (2008). IL-20 may contribute to the pathogenesis of human intervertebral disc herniation. Spine.

[16] Hsu Y.-H., Li H.-H., Hsieh M.-Y., Liu M.-F., Huang K.-Y., Chin L.-S., Chen P.-C., Cheng H.-H., Chang M.-S. (2006). Function of interleukin-20 as a proinflammatory molecule in rheumatoid and experimental arthritis. Arthritis Rheum..

[17] Hsieh M.-Y., Chen W.-Y., Jiang M.-J., Cheng B.-C., Huang T.-Y., Chang M.-S. (2006). Interleukin-20 promotes angiogenesis in a direct and indirect manner. Genes Immun..

[18] Li A., Dubey S., Varney M.L., Dave B.J., Singh R.K. (2003). IL-8 directly enhanced endothelial cell survival, proliferation, and matrix metalloproteinases production and regulated angiogenesis. J. Immunol..

[19] Ha H.-L., Wang H., Claudio E., Tang W., Siebenlist U. (2019). IL-20-Receptor Signaling Delimits IL-17 Production in Psoriatic Inflammation. J. Investig. Dermatol..

[20] Sabat R., Wolk K. (2011). Research in practice: IL-22 and IL-20: Significance for epithelial homeostasis and psoriasis pathogenesis. J. der Dtsch. Dermatol. Ges..

[21] Chan J.R., Blumenschein W., Murphy E., Diveu C., Wiekowski M., Abbondanzo S., Lucian L., Geissler R., Brodie S., Kimball A.B. (2006). IL-23 stimulates epidermal hyperplasia via TNF and IL-20R2-dependent mechanisms with implications for psoriasis pathogenesis. J. Exp. Med..

[22] Blumberg H., Conklin D., Xu W., Grossmann A., Brender T., Carollo S., Eagan M., Foster D., Haldeman B.A., Hammond A. (2001). Interleukin 20: Discovery, receptor identification, and role in epidermal function. Cell.

[23] Commins S., Steinke J., Borish L. (2008). The extended IL-10 superfamily: IL-10, IL-19, IL-20, IL-22, IL-24, IL-26, IL-28, and IL-29. J. Allergy Clin. Immunol..

[24] Myles I., Fontecilla N.M., Valdez P.A., Vithayathil P.J., Naik S., Belkaid Y., Ouyang W., Datta S. (2013). Signaling via the IL-20 receptor inhibits cutaneous production of IL-1β and IL-17A to promote infection with methicillin-resistant Staphylococcus aureus. Nat. Immunol..

[25] Tritsaris K., Myren M., Ditlev S.B., Hübschmann M.V., van der Blom I., Hansen A.J., Olsen U.B., Cao R., Zhang J., Jia T. (2007). IL-20 is an arteriogenic cytokine that remodels collateral networks and improves functions of ischemic hind limbs. Proc. Natl. Acad. Sci. USA.

[26] Waszczykowski M., Fabiś-Strobin A., Bednarski I., Narbutt J., Fabiś J. (2022). Serum and synovial fluid concentrations of interleukin-18 and interleukin-20 in patients with osteoarthritis of the knee and their correlation with other markers of inflammation and turnover of joint cartilage. AMS.

[27] Šenolt L., Prajzlerová K., Hulejová H., Šumová B., Filková M., Veigl D., Pavelka K., Vencovský J. (2017). Interleukin-20 is triggered by TLR ligands and associates with disease activity in patients with rheumatoid arthritis. Cytokine.

[28] Valentina M., Jan F., Peder N.L., Bo Z., Hongjie D., Pernille K. (2015). Cytokine detection and simultaneous assessment of rheumatoid factor interference in human serum and synovial fluid using high-sensitivity protein arrays on plasmonic gold chips. BMC Biotechnol..

[29] Hsu Y.-H., Chen W.-Y., Chan C.-H., Wu C.-H., Sun Z.-J., Chang M.-S. (2011). Anti-IL-20 monoclonal antibody inhibits the differentiation of osteoclasts and protects against osteoporotic bone loss. J. Exp. Med..

[30] Hsu Y.-H., Hsing C.-H., Li C.-F., Chan C.-H., Chang M.-C., Yan J.-J. (2012). Anti-IL-20 monoclonal antibody suppresses breast cancer progression and bone osteolysis in murine models. J. Immunol..

[31] Yamaguchi M., Fukasawa S. (2021). Is Inflammation a Friend or Foe for Orthodontic Treatment?: Inflammation in Orthodontically Induced Inflammatory Root Resorption and Accelerating Tooth Movement. Int. J. Mol. Sci..

[32] Antoun J.S., Mei L., Gibbs K., Farella M. (2017). Effect of orthodontic treatment on the periodontal tissues. Periodontology 2000.

[33] Li Y., Zhan Q., Bao M., Yi J., Li Y. (2021). Biomechanical and biological responses of periodontium in orthodontic tooth movement: Up-date in a new decade. Int. J. Oral Sci..

[34] Meng B., Wu D., Cheng Y., Huang P., Liu Y., Gan L., Liu C., Cao Y. (2020). Interleukin-20 differentially regulates bone mesenchymal stem cell activities in RANKL-induced osteoclastogenesis through the OPG/RANKL/RANK axis and the NF-κB, MAPK and AKT signalling pathways. Scand. J. Immunol..

[35] Yang B., Fu C., Wu Y., Liu Y., Zhang Z., Chen X., Wu D., Gan Z., Chen Z., Cao Y. (2022). γ-Secretase inhibitors suppress IL-20-mediated osteoclastogenesis via Notch signalling and are affected by Notch2 in vitro. Scand. J. Immunol..

[36] Singh S., Anshita D., Ravichandiran V. (2021). MCP-1: Function, regulation, and involvement in disease. Int. Immunopharmacol..

[37] Otero K., Turnbull I., Poliani P., Vermi W., Cerutti E., Aoshi T., Tassi I., Takai T., Stanley S., Miller M. (2009). Macrophage colony-stimulating factor induces the proliferation and survival of macrophages via a pathway involving DAP12 and beta-catenin. Nat. Immunol..

[38] Yoshida H., Hayashi S.-I., Kunisada T., Ogawa M., Nishikawa S., Okamura H., Sudo T., Shultz L.D., Nishikawa S.-I. (1990). The murine mutation osteopetrosis is in the coding region of the macrophage colony stimulating factor gene. Nature.

[39] Zur Y., Rosenfeld L., Keshelman C., Dalal N., Guterman-Ram G., Orenbuch A., Einav Y., Levaot N., Papo N. (2018). A dual-specific macrophage colony-stimulating factor antagonist of c-FMS and alphavbeta3 integrin for osteoporosis therapy. PLoS Biol..

[40] Wiktor-Jedrzejczak W., Gordon S. (1996). Cytokine regulation of the macrophage (M phi) system studied using the colony stimulating factor-1-deficient op/op mouse. Physiol. Rev..

[41] Liu L., Ding C., Zeng W., Heuer J.G., Tetreault J.W., Noblitt T.W., Hangoc G., Cooper S., Brune K.A., Sharma G. (2003). Selective enhancement of multipotential hematopoietic progenitors in vitro and in vivo by IL-20. Blood.

[42] Rutz S., Wang X., Ouyang W. (2014). The IL-20 subfamily of cytokines--from host defence to tissue homeostasis. Nat. Rev. Immunol..

[43] Zhang W., Magadi S., Li Z., Smith C.W., Burns A.R. (2017). IL-20 promotes epithelial healing of the injured mouse cornea. Exp. Eye Res..

[44] Ikebuchi Y., Aoki S., Honma M., Hayashi M., Sugamori Y., Khan M., Kariya Y., Kato G., Tabata Y., Penninger J.M. (2018). Coupling of bone resorption and formation by RANKL reverse signalling. Nature.

[45] Coury F., Peyruchaud O., Machuca-Gayet I. (2019). Osteoimmunology of Bone Loss in Inflammatory Rheumatic Diseases. Front. Immunol..

[46] Ralston S., Schett G. (2018). Osteoimmunology. Calcif. Tissue Int..

[47] Okamoto K., Takayanagi H. (2019). Osteoimmunology. Cold Spring Harb. Perspect. Med..

[48] Xiong J., Onal M., Jilka R.L., Weinstein R.S., Manolagas S.C., O’Brien C.A. (2011). Matrix-embedded cells control osteoclast formation. Nat. Med..

[49] Cao X. (2011). Targeting osteoclast-osteoblast communication. Nat. Med..

[50] Theill L.E., Boyle W.J., Penninger J.M. (2022). RANK-L and RANK: T cells, bone loss, and mammalian evolution. Annu. Rev. Immunol..

[51] Danks L., Komatsu N., Guerrini M.M., Sawa S., Armaka M., Kollias G., Nakashima T., Takayanagi H. (2015). RANKL expressed on synovial fibroblasts is primarily responsible for bone erosions during joint inflammation. Ann. Rheum. Dis..

[52] Boyle W.J., Simonet W.S., Lacey D.L. (2003). Osteoclast differentiation and activation. Nature.

[53] Lechner J., Rudi T., von Baehr V. (2018). Osteoimmunology of tumor necrosis factor-alpha, IL-6, and RANTES/CCL5: A review of known and poorly understood inflammatory patterns in osteonecrosis. Clin. Cosmet. Investig. Dent..

[54] Mansky K.C., Sankar U., Han J., Ostrowski M.C. (2002). Microphthalmia transcription factor is a target of the p38 MAPK pathway in response to receptor activator of NF-kappa B ligand signaling. J. Biol. Chem..

[55] Kim T., Yoon J., Cho H., Lee W.B., Kim J., Song Y.H., Kim S.N., Yoon J.H., Kim-Ha J., Kim Y.J. (2005). Downregulation of lipopolysaccharide response in Drosophila by negative crosstalk between the AP1 and NF-kappaB signaling modules. Nat. Immunol..

[56] Seeman E., Delmas P.D. (2006). Bone quality--the material and structural basis of bone strength and fragility. N. Engl. J. Med..

[57] Lacey D.L., Boyle W.J., Simonet W.S., Kostenuik P.J., Dougall W.C., Sullivan J.K., Martin J.S., Dansey R. (2012). Bench to bedside: Elucidation of the OPG-RANK-RANKL pathway and the development of denosumab. Nat. Rev. Drug Discov..

[58] Nishida H., Suzuki H., Madokoro H., Hayashi M., Morimoto C., Sakamoto M., Yamada T. (2014). Blockade of CD26 signaling inhibits human osteoclast development. J. Bone Miner. Res. Off. J. Am. Soc. Bone Miner. Res..

[59] Zhu M., Sun B.H., Saar K., Simpson C., Troiano N., Dallas S.L., Tiede-Lewis L.M., Nevius E., Pereira J.P., Weinstein R.S. (2016). Deletion of Rac in Mature Osteoclasts Causes Osteopetrosis, an Age-Dependent Change in Osteoclast Number, and a Reduced Number of Osteoblasts In Vivo. J. Bone Miner. Res. Off. J. Am. Soc. Bone Miner. Res..

[60] Su N., Li X., Tang Y., Yang J., Wen X., Guo J., Tang J., Du X., Chen L. (2016). Deletion of FGFR3 in Osteoclast Lineage Cells Results in Increased Bone Mass in Mice by Inhibiting Osteoclastic Bone Resorption. J. Bone Miner. Res. Off. J. Am. Soc. Bone Miner. Res..

[61] Wu D., Meng B., Cheng Y., Gan L., Huang P., Cao Y. (2019). The effect of risedronate on orthodontic tooth movement in ovariectomized rats. Arch. Oral Biol..

[62] Hsu Y.-H., Chiu Y.-S., Chen W.-Y., Huang K.-Y., Jou I.-M., Wu P.-T., Wu C.-H., Chang M.-S. (2016). Anti-IL-20 monoclonal antibody promotes bone fracture healing through regulating IL-20-mediated osteoblastogenesis. Sci. Rep..

[63] Chen X., Liu Y., Meng B., Wu D., Wu Y., Cao Y. (2021). Interleukin-20 inhibits the osteogenic differentiation of MC3T3-E1 cells via the GSK3β/β-catenin signalling pathway. Arch. Oral Biol..

[64] Dai Q., Han Y., Xie F., Ma X., Xu Z., Liu X., Zou W., Wang J. (2018). A RANKL-based Osteoclast Culture Assay of Mouse Bone Marrow to Investigate the Role of mTORC1 in Osteoclast Formation. J. Vis. Exp..

[65] Marino S., Logan J.G., Mellis D., Capulli M. (2014). Generation and culture of osteoclasts. BoneKEy Rep..

