# Supplementary material for: Dual Role of Interleukin-20 in Different Stages of Osteoclast Differentiation and Its Osteoimmune Regulation during Alveolar Bone Remodeling
Source: Int J Mol Sci. 2023 Feb 14;24(4):3810. doi: 10.3390/ijms24043810 (PMC9961846; doi:10.3390/ijms24043810)
Supplement: Supplementary file 1 [file ijms-24-03810-s001.zip › ijms-2099354-supplementary.pdf]

Dual Role of Interleukin-20 in Different Stages of Osteoclast Differentiation and Its Osteoimmune Regulation during Alveolar Bone Remodeling

Bowen Meng [1,2,3,†], Benyi Yang [1,2,3,†], Yan Qu [1,2,3], Yuanbo Liu [1,2,3], Dongle Wu [1,2,3], Chaoran Fu [1,2,3], Yifan He [1,2,3], Xi Chen [1,2,3], Chufeng Liu [4], Xiaoxing Kou [1,2,3,*] and Yang Cao [1,2,3,*]

1 Hospital of Stomatology, Sun Yat-sen University, Guangzhou 510055, China.

2 Guangdong Provincial Key Laboratory of Stomatology, Guangzhou 510055, China.

3 South China Center of Craniofacial Stem Cell Research, Guanghua School of Stomatology, Sun Yat-sen University, Guangzhou 510055, China

4 Department of Orthodontics, Stomatological Hospital, Southern Medical University, No.366 Jiangnan Da Dao South Haizhu District, Guangzhou 510260, China

* Correspondence:
kouxiaoxing@mail.sysu.edu.cn (X.K.); caoyang@mail.sysu.edu.cn (Y.C.)

Correspondences
Prof. Yang Cao, Hospital of Stomatology, Guanghua School of Stomatology, Sun Yat-sen University, Guangdong Provincial Key Laboratory of Stomatology, Guangzhou 510055, China.
Tel: +8613922278766
Email: caoyang@mail.sysu.edu.cn
Prof. Xiaoxing Kou, South China Center of Craniofacial Stem Cell Research, Hospital of Stomatology, Guanghua School of Stomatology, Sun Yat-sen University, Guangdong Provincial Key Laboratory of Stomatology, Guangzhou 510055, China.
Tel: +8613581814339
Email: kouxiaoxing@mail.sysu.edu.cn
† These authors contributed equally to this work.

Supplementary Figures and figure legends

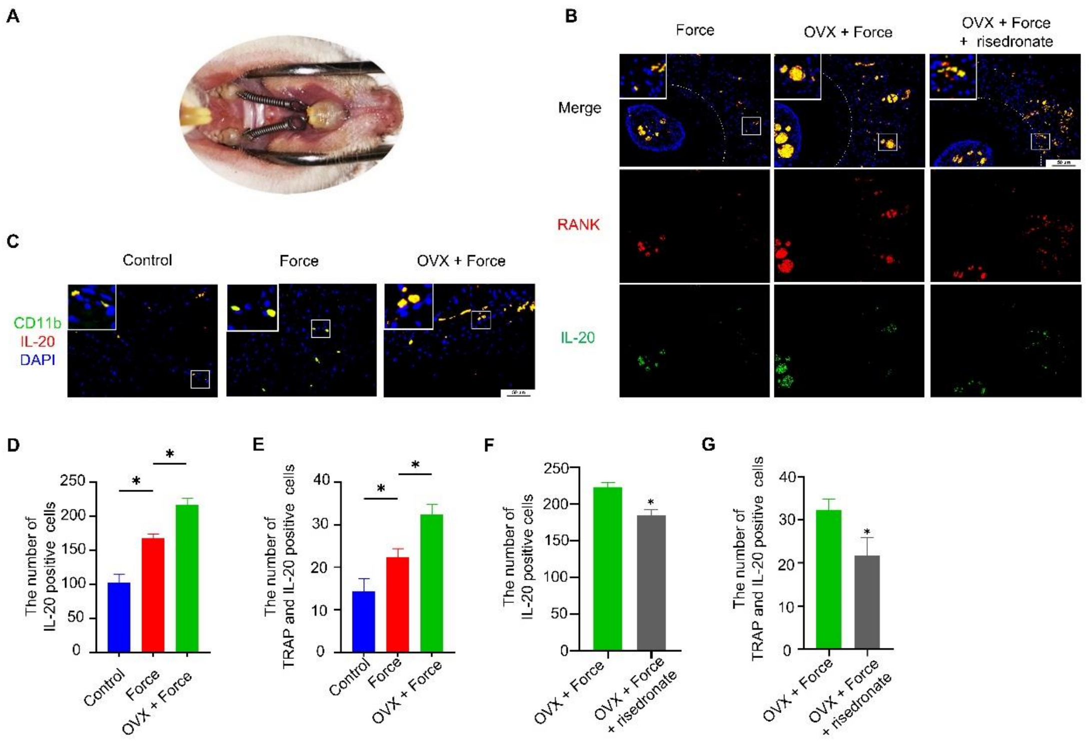

Figure S1. IL-20 accelerated orthodontic tooth movement. **(A)** The rat model of orthodontic tooth movement. **(B)** Double-labelled immunofluorescence staining showed that, in the context of orthodontic force, the expression levels of IL-20 and osteoclast marker protein RANK increased in the first molar periodontal ligament. **(C)** Immunofluorescence staining showed that the expression levels of IL-20 and CD11b in the first molar periodontal ligament after the application of orthodontic force. **(D)** The statistical analysis of IL-20 positive cells in the Control group, Force group, and OVX + Force group. **(E)** The statistical analysis of TRAP and IL-20 positive cell number of the first molar periodontal ligament in the Control group, Force group and OVX + Force group. **(F)** The statistical analysis of IL-20 positive cells in the OVX + Force group and OVX + Force + risedronate group. **(G)** The statistical analysis of TRAP and IL-20 positive cell number of the first molar periodontal ligament in the OVX + Force group and OVX + Force + risedronate group. *$p < 0.05$ vs. the control group. $n = 6$.

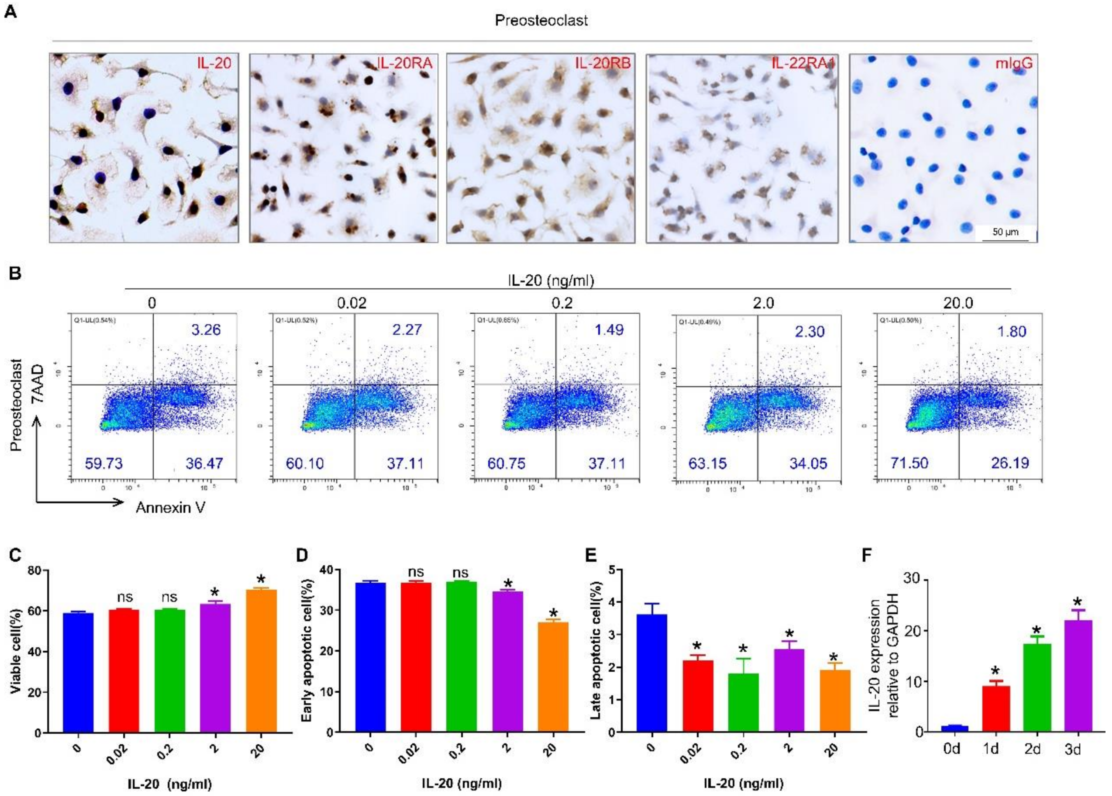

Figure S2. IL-20 inhibited preosteoclast apoptosis. **(A)** The expression of IL-20, IL-20RA, IL-20RB, IL-22RA1 and isotype control (mIgG) in M-CSF-induced preosteoclasts was determined by immunochemical staining. **(B)** Cell apoptosis were examined in M-CSF-induced preosteoclasts by flow cytometry assay. **(C-E)** Apoptotic cells were detected using an Annexin V-APC/7-AAD apoptosis kit after 3 days of IL-20 treatment. **(F)** The mRNA expression level of IL-20 in BMMs was evaluated by qRT-PCR after 30 ng/ml M-CSF treatment. * $p < 0.05$ vs. control group. ns $p > 0.05$ vs. control group. $n = 6$.

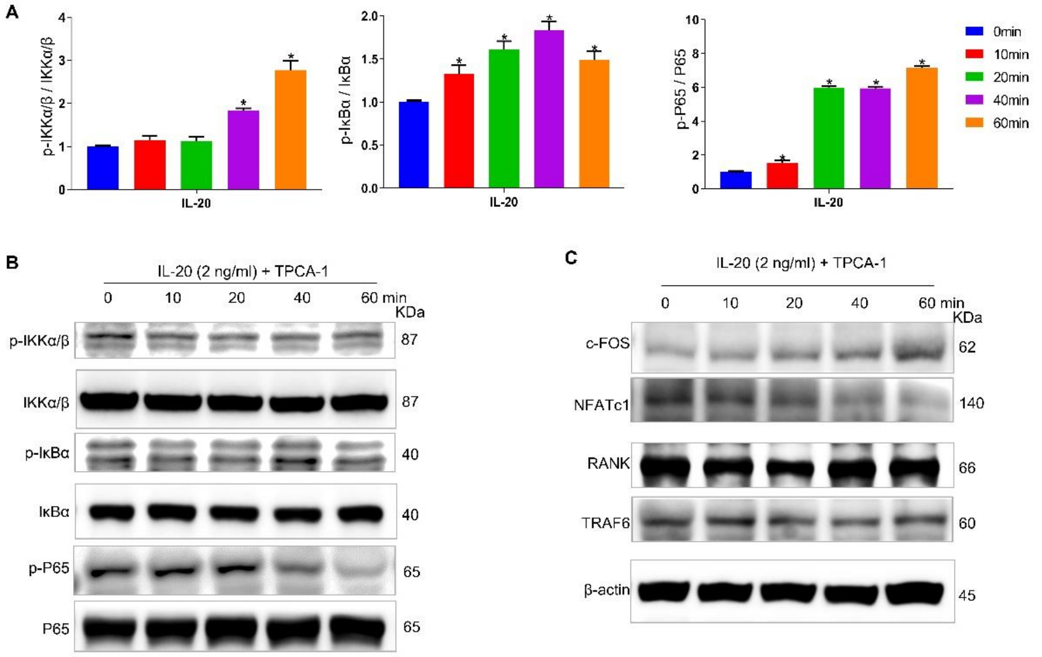

Figure S3. IL-20-mediated activation of NF-κB pathway was blocked by TPCA-1. Preosteoclasts were stimulated with IL-20 and NF-κB pathway inhibitor TPCA-1. **(A)** The levels of phosphorylation for proteins in the NF-κB pathway, including the IKKα/β, IκB-α, and P65 proteins in IL-20 treated preosteoclasts, were detected using Western blotting. **(B)** The levels of phosphorylation for proteins in the NF-κB pathway, including the IKKα/β, IκB-α, and P65 proteins in IL-20 + TPCA-1 treated preosteoclasts, were detected using Western blotting. **(C)** The levels of activated proteins in signaling pathways, including the RANK, TRAF6, c-Fos and NFATc1 proteins without RANKL, were detected using Western blotting. * $p < 0.05$ vs. the 0 ng/ml IL-20 group. $n = 6$.

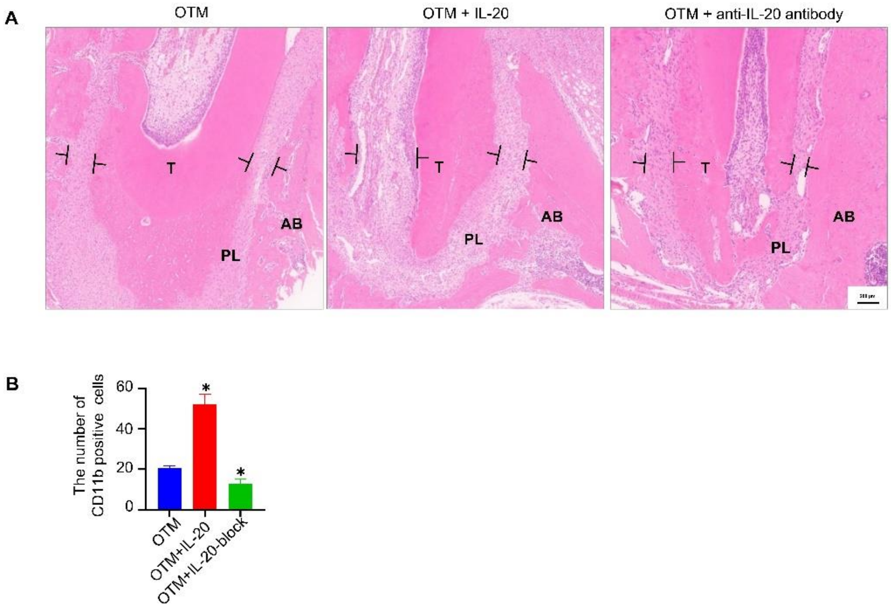

Figure S4. The effect of IL-20 on orthodontic tooth movement and osteoclast differentiation. **(A)** HE staining showed significant changes in first molar periodontal ligament thickness. **(B)** Immunofluorescence staining showed that the expression levels of MCP-1 and CD11b in the first molar periodontal ligament after the application of orthodontic force. OTM + IL-20-block group meant that rats were locally infused with anti-IL-20 antibody. * $p < 0.05$ vs. the control group. $n = 6$.

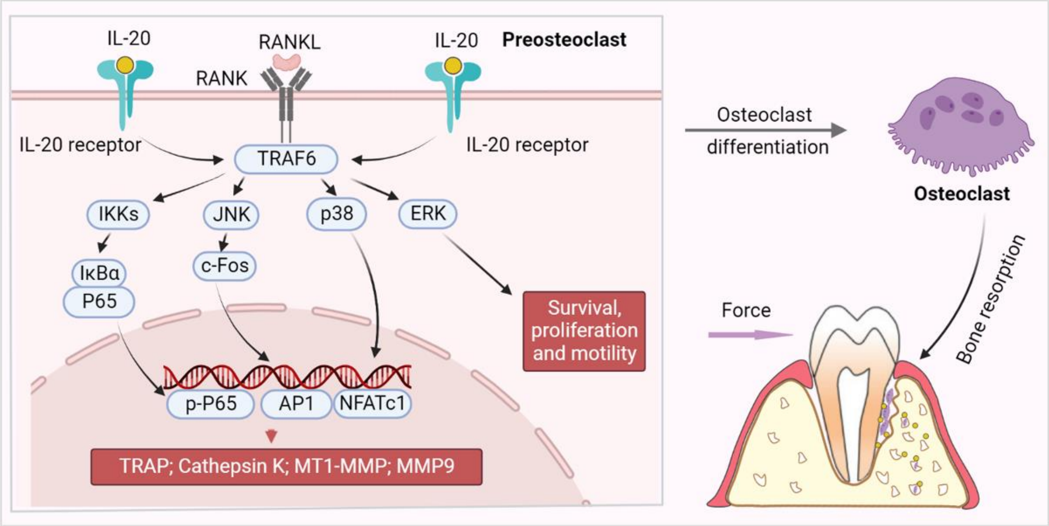

Figure S5. A schematic diagram shows the IL-20 – mediated regulation of osteoclast formation and function through the NF-κB and MAPK signaling pathways.

中山大學 SUN YAT-SEN UNIVERSITY

中山大学实验动物管理与使用委员会
Institutional Animal Care and Use Committee,
Sun Yat-Sen University

中山大学动物实验伦理审查同意书
Affidavit of Approval of Animal Use Protocol, IACUC, SYSU

申请编号 Application No.	2018000294	批准编号 Approval No.	SYSU-IACUC-2018-000099

本动物实验方案经过中山大学实验动物伦理委员会审核，符合动物保护、动物福利和伦理原则，符合国家实验动物福利伦理的相关规定。The animal use protocol listed below has been reviewed and approved by the Institutional Animal Care and Use Committee (IACUC), Sun Yat-Sen University.

实验名称 Protocol Title	利塞膦酸钠对卵巢切除 SD 大鼠正畸牙移动的影响和机制研究<br>The effect and mechanism of risedronate sodium in OVX SD rats following orthodontic tooth movement				
实验申请人 Applicant	吴冬乐<br>Donald	职称/学位 Title/Degree	硕士<br>Master	邮箱 Email	wudongle@qq.com
实验负责人 Principle Investigator (PI)	曹阳<br>Yang Cao	职称/学位 Title/Degree	教授<br>Professor	邮箱 Email	caoyang34@163.com
院系(部门) Department	中山大学光华口腔医学院<br>Guanghua School of stomatology, Hospital of stomatology, Sun Yat-sen University	申请日期 Application date	2018/7/12		
动物种系 Species or Strains	大鼠 CD(SD)IGS<br>CD(SD)IGS	动物数量 Quantity	75		
计划执行时间 Period of Protocol	2018/7/13 ~2020/7/13	实验动物使用许可证 Number of Animal use permit	中山大学（实验动物中心北校园）（SYXK（粤）2017-0081）		
审查意见 Results of inspection	☑符合动物福利伦理要求，同意实验 Agree<br>☐调整方案后，可进行实验 Agree after modification				
兽医师 Chief Veterinary Officer	[illegible]	日期 Date	2019. 10. 25		

中山大学实验动物伦[illegible]UC，SYSU）
主席（Chairma[illegible]
日期（Date）：201[illegible]

中山大学实验动物管理与使用委员会 IACUC·SYSU

[illegible]

地址：广州中山二路 74 号中山大学实验动物中心 邮编：510080
Add: Laboratory Animal Center, SUN YAT-SEN UNIVERSITY, No. 74, Zhongshan Road II, Guangzhou, 510080, P.R. China

第 1 页，共 1 页
